# Deficits in Early Sensory and Cognitive Processing Are Related to Phase and Nonphase EEG Activity in Multiple Sclerosis Patients

**DOI:** 10.3390/brainsci11050629

**Published:** 2021-05-13

**Authors:** Esteban Sarrias-Arrabal, Sara Eichau, Alejandro Galvao-Carmona, Elvira Domínguez, Guillermo Izquierdo, Manuel Vázquez-Marrufo

**Affiliations:** 1 Experimental Psychology Department, Faculty of Psychology, University of Seville, 41018 Seville, Spain; marrufo@us.es; 2 Unit CSUR Multiple Sclerosis, Hospital Virgen Macarena, 41009 Seville, Spain; saraeichau@gmail.com; 3 Departamento de Psicología, Universidad Loyola Andalucía, 41704 Sevilla, Spain; agalvao@uloyola.es; 4 Unit of Multiple Sclerosis, FISEVI, Hospital Virgen Macarena, 41009 Seville, Spain; elvira.dominguez@juntadeandalucia.es; 5 Unit of Multiple Sclerosis, Hospital VITHAS, 41950 Seville, Spain; guillermo.izquierdo@fundaciondinac.com

**Keywords:** alpha, oddball, EEG, gamma, multiple sclerosis, evoked, induced

## Abstract

Currently, there is scarce knowledge about the relation between spectral bands modulations and the basis of cognitive impairment in multiple sclerosis (MS). In this sense, analyzing the evoked or phase activity can confirm results from traditional event-related potential (ERP) studies. However, studying the induced or nonphase activity may be necessary to elucidate hidden compensatory or affected cognitive mechanisms. In this study, 30 remitting-relapsing multiple sclerosis patients and 30 healthy controls (HCs) matched in sociodemographic variables performed a visual oddball task. The main goal was to analyze phase and nonphase alpha and gamma bands by applying temporal spectral evolution (TSE) and its potential relation with cognitive impairment in these patients. The behavioural results showed slower reaction time and poorer accuracy in MS patients compared to controls. In contrast, the time-frequency analysis of electroencephalography (EEG) revealed a delay in latency and lower amplitude in MS patients in evoked and induced alpha compared to controls. With respect to the gamma band, there were no differences between the groups. In summary, MS patients showed deficits in early sensorial (evoked alpha activity) and cognitive processing (induced alpha activity in longer latencies), whereas the induced gamma band supported the hypothesis of its role in translation of attentional focus (induced activity) and did not show strong activity in this paradigm (visual oddball).

## 1. Introduction

Multiple sclerosis (MS) is a neurodegenerative disease of unknown etiology, in which the main neurological damage is demyelination and inflammation of the central nervous system (CNS) [1]. MS is a disease predominant in women, and its onset occurs over a wide age range (mainly between 20 and 40 years) [2]. Moreover, MS shows diverse cognitive deficits in 40–70% of cases [1], and different neuropsychological profiles have been found by clinical assessments. Diverse studies have defined that attention, processing speed and memory are the cognitive domains most frequently affected [3,4].

One of the cognitive paradigms that has been used to understand the neural basis of these cognitive alterations in MS is the oddball task. Most studies have found a delay in the latency of event-related potentials (ERPs) post-stimulus (mismatch negativity, N1, P1, P2 and P300) [5,6,7]. However, some authors have not described differences in ERPs between groups [8]. In addition, the oddball task can be a useful tool because changes in ERPs correlate with changes in scores of neuropsychological tests [9,10] and magnetic resonance imaging (MRI) parameters [6,11]. Even ERPs obtained while performing visual oddball tasks predict cognitive functioning and processing speed in patients with MS [12].

The combined use of spectral analysis and the oddball task has been applied in patients with MS [13], resulting in an increased power in beta and gamma bands. This increase is associated with fronto-cortical atrophy as suggested by other authors [6,14]. However, these studies have only analyzed the frequency domain without the time dimension. To improve our knowledge of the neural basis of cognitive impairment in MS pathology, the time-frequency domain of the electroencephalography (EEG) signals should be analyzed to add temporal information to frequency domain methods. To the best of our knowledge, the time-frequency domain has never been studied in MS when applying auditory or visual oddball tasks.

Prior to focusing on MS, it is important to describe the relation between the time-frequency features of spectral EEG bands and specific cognitive mechanisms. With respect to the analyzed bands in this study (alpha and gamma), there is considerable literature related to their functional role. The alpha band was originally proposed as an indicator of a neural area at rest [15]; however, several authors have subsequently suggested that it can also play an active role in inhibitory control and timing of sensory processing [16]. In this sense, the increase (synchronization) in alpha activity in regions irrelevant to the task has been associated with inhibition processes [17]. Additionally, some studies have proposed that the synchronization of alpha in latencies of ERPs (P1 and N1) [18,19] represents the early sensorial processing of stimulus [20]. In contrast, the decrease (desynchronization) in the alpha band has also been related to psychophysiological roles [21]. Specifically, a decrease in alpha has been associated with general attention and specific processes, such as semantic processing [22,23,24,25], which indicates that a larger decrease (desynchronization) in alpha activity leads to better behavioural performance.

With regard to gamma activity, researchers have related such activity to several cognitive processes [26,27]. However, a diverse range of gamma activities has been studied, which has resulted in discrepancies. These contradictory results may be based on different cognitive demands, tasks and analyzed ranges. The gamma band has been fundamentally associated with visual binding [28,29,30], spatial attention [31], processing target stimuli [32] or translation of attentional focus [20,33]. One of the reasons that makes this band suitable for attentional mechanisms is its fine temporal tuning for neuronal firing (10–30 ms time precision) [34].

With respect to these spectral bands, most studies have analyzed the alpha band in patients with MS with similar conclusions. These studies have suggested that a smaller decrease in the alpha band for MS patients compared to healthy controls is associated with a poorer behavioural performance [13,35,36]. However, a larger decrease (desynchronization) in alpha activity in patients with MS has been described in certain conditions that demands more cognitive resources [20]. These authors have suggested that in some conditions, MS patients need a larger decrease in the alpha band to improve cognitive processing (compensatory mechanism).

In the case of the gamma band, to the best of our knowledge, few studies have described alterations in this band in MS patients. It has been proposed that alterations in the gamma band may be related to deficits in cognitive processing [37]. More specifically, an increase (synchronization) in the gamma band in patients with MS compared to healthy controls has been associated with plasticity or compensatory attentional mechanisms [20,38].

As suggested above, time-frequency methods are better than frequency methods for understanding the role of spectral bands. One of these time-frequency techniques is temporal spectral evolution (TSE) [39]. The TSE allows EEG time-frequency information that includes both evoked (phase) and induced (nonphase) modulations for a spectral band related to a stimulus presentation to be obtained (for a detailed description of the TSE method, see [40,41,42]). Both evoked and induced activities have been demonstrated to represent different cognitive processes [20].

Finally, TSE has rarely been applied in patients with MS to observe spectral modulations. Applying the attention network test (ANT), a lower increase (synchronization) in amplitude in evoked (phase) alpha activity and a delayed latency in induced (nonphase) alpha activity have been described in MS patients [20]. Regarding the gamma band, a larger induced (non-phase) gamma activity has been found in MS patients compared to healthy controls [20]. Alterations in alpha bands have been associated with deficits in early sensory and cognitive processing, and changes in gamma bands have been associated with deficits in translation of attentional focus.

Considering these premises, we hypothesized that the alpha band would be altered in the MS group even with an oddball task where the cognitive mechanisms involved are simpler than in the attention network test (ANT) [20]. We also hypothesized that the gamma band would not show differences between the two groups because attention focus does not change throughout the experiment (central presentation). Therefore, the main aims of the present study were to analyze the potential modulations in alpha and gamma bands (evoked and induced), as well as to relate them to cognitive impairment in MS patients.

## 2. Methods

### 2.1. Participants

Sixty subjects were enrolled to perform the experiment in controlled conditions. Thirty patients (22 women and 8 men) with relapsing-remitting MS (RRMS) were recruited from the Multiple Sclerosis Unit of the Hospital Universitario Virgen Macarena (Seville, Spain). The patients were between the ages of 27 and 59 years (mean 41.5, standard deviation (SD) 9.04). A healthy control (HC) group with thirty subjects was selected with sociodemographic variables matched to the MS group (19 women and 11 men who were between the ages of 24 and 52 years (mean 37.43, SD 10.68) (Table 1). Each group had three subjects who were left-handed.

Regarding patients with RRMS, the definite diagnosis was made by a neurologist according to McDonald’s criteria [43]. To be eligible for inclusion in the study, patients were required to be under an Expanded Disability Status Scale (EDSS) of 5.5 (mean 2.95, SD 1.6). The following exclusion criteria were used for this study: clinical relapses in the last month; presence of comorbid neurodegenerative or psychiatric disorders; severe signs of depression; history of substance abuse; head trauma; vascular diseases and seizures; significant upper limb impairment; or visual acuity or field deficits. The mean duration of the disease (in years) and the standard deviation were 8.46 and 4.36, respectively. All healthy subjects were declared to be free of neurological conditions.

This study was performed in compliance with the Helsinki Declaration. All participants signed informed consent before their inclusion, and the protocol was approved by the Ethics Committee of the University of Seville (project code: PSI2016–78133-P).

### 2.2. Cognitive Task

Participants were seated in a sound-attenuated room in front of a computer monitor. Stimuli were created by E-prime 2.0 (Psychology Software Tools, Inc., Pittsburgh, PA, USA) and presented on a liquid crystal display (LCD) screen. The cognitive task used was a “visual oddball” in which the subject had to discriminate uncommon visual stimuli (target) (probability: 25%) in a sequence of frequent stimuli (standard). The target stimulus consisted of a rectangle with a checkerboard pattern that was comprised of red and white squares. The standard (frequent) stimulus was equivalent in size with the same pattern but with black and white squares. All stimuli subtended a 7.98° × 9.42° visual angle at a viewing distance of 80 cm. Both stimuli were displayed randomly in the center of the screen. When a target was displayed, the subject was required to press the mouse button with the right index finger and ignore the standard stimulus. All stimuli were presented for 500 ms, and the stimulus onset asynchrony (SOA) was 1 s, during which the subject could respond. A fixation point was present during the SOA to avoid changes in eye position during the experiment. One block with 200 trials was used to obtain good performance in a target/standard task. At the end of the experimental session, the reaction time and percentage accuracy (including no responses for the standard stimuli) were calculated. All participants were asked to respond as quickly and accurately as possible.

### 2.3. Electroencephalography (EEG) Recording and Analyses

**Figure 1:**
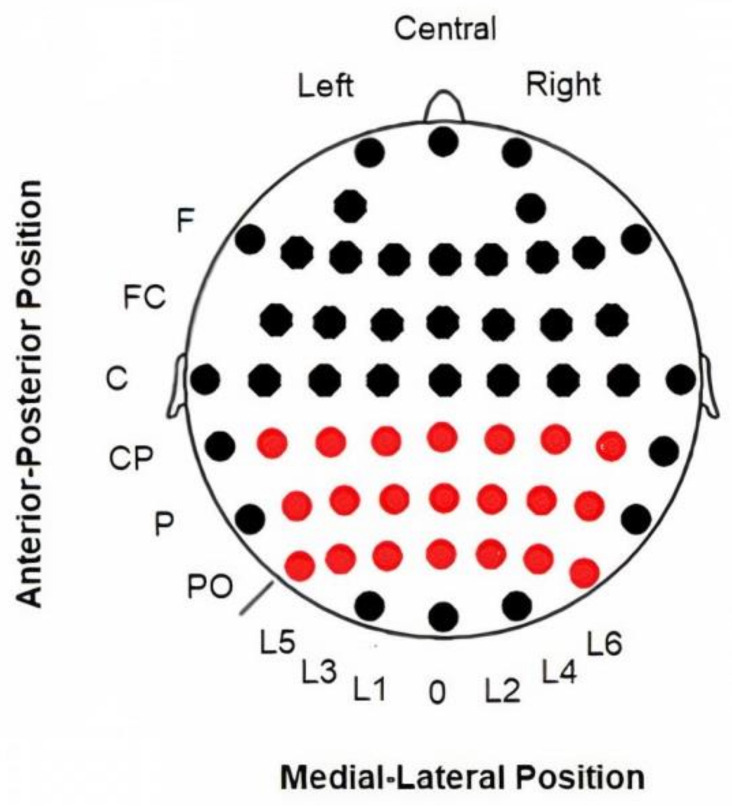
Electrode matrix selected to analyze spectral activity.

EEG data were recorded from 58 electrodes (Ag/AgCl) in standard locations of a 10–10 system [44] and amplified with BrainAmp amplifiers (Brain Products GmbH, Gilching, Germany) The detailed positions of electrodes are shown in Figure 1. The EEG signal was filtered online with a bandpass of 0.01 to 100 Hz, digitized with a sampling rate of 500 Hz and stored using Brain Vision Recorder software (Brain Products GmbH, Germany). All continuous data were referenced online to the linked auricular lobes and offline to a common averaged reference. Impedance was kept below 5 kOhm during the experiment. Vertical electrooculograms (VEOGs) and horizontal electrooculograms (HEOGs) were also recorded with a bipolar montage. Trials with a HEOG signal outside the ±50 µV range were rejected. For blinking artefacts, ocular correction was performed in the scalp electrodes using the algorithm developed by the authors [45]. A continuous signal was epoched in segments of −1000 to 1000 ms with zero being the onset of the target. A baseline correction (−200 to 0 ms) was also applied to both conditions. We segmented the signal in intervals of 2000 ms to avoid edge artefacts in the spectral modulations studied [46].

**Figure 2:**
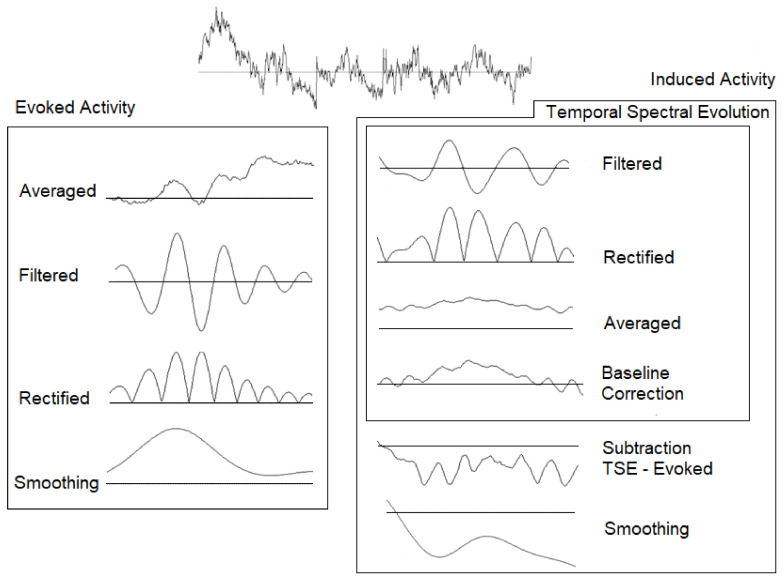
Temporal spectral evolution (TSE).

After processing, the following two possible analyses were performed for the EEG signal: the target condition was averaged independently to obtain evoked activity, and the signal was filtered for alpha (8–13 Hz) and gamma (30–45 Hz) bands and rectified [47]. Prior to the induced activity, the temporal spectral evolution (TSE) method was calculated with the following steps: (1) identical bandpass filtering in previously defined alpha and gamma bands was performed over the EEG epochs, (2) the signal was rectified, (3) the target stimuli were averaged, and (4) a baseline correction (−200 to 0 ms) was also applied. After this analysis, a subtraction of evoked activity from the TSE was subsequently performed to calculate the induced response (nonphase activity) [47] (Figure 2).

Following the guidelines proposed by Keil and Müller [48], the latency was calculated at the electrode with the maximum amplitude (p4p) in the average of the target condition in both groups. The latency peak was determined individually for each participant. Moreover, the amplitude was analyzed at different intervals for evoked and induced activity after the onset stimulus. For alpha activity, the amplitude in the evoked activity was exported for the interval of 125–155 ms, while for the induced activity, the interval was 120–220 ms. These intervals included the latency at which both groups reached their maximum amplitude values. In addition, the mean amplitude value in the interval of 220–800 ms was exported in the induced activity because both groups showed a decrease in induced alpha activity after the first valley observed in the target condition. For gamma activity, the amplitude in the evoked activity was exported for the interval of 90–160 ms, while for the induced activity, the interval was 90–130 ms. All the intervals were identical for both groups. The mean amplitude values were exported for the entire interval in a matrix of 3 × 7 electrodes that covered the posterior area of the scalp in both bands (Figure 1).

### 2.4. Statistical Analyses

#### 2.4.1. Behavioural Responses

The Shapiro–Wilk test (*p* > 0.05) was applied to check for normality. Parametric (analysis of variance, ANOVA) or non-parametric tests (Mann–Whitney U test) were performed to study possible differences in reaction time or accuracy. The analysis of the reaction times was performed with a single-factor ANOVA using the following factor: group factor (levels: HC and MS patients). The accuracy of the subjects’ responses was determined with the Mann–Whitney U test with the same factor as for the reaction time ANOVA.

#### 2.4.2. Alpha Band

In the case of the analysis of alpha activity, evoked and induced latencies were analyzed together. One ANOVA with two variables was performed with the following factors: group factor (levels HC and MS patients) and activity factor (levels: evoked and induced activity). Regarding amplitude, evoked activity of the 125–155 ms interval was analyzed with ANOVA with the following factors: group factor (levels: HC and MS patients); anterior-posterior factor (levels: mid-parietal, parietal and parietal-posterior); and lateral-medial factor (line 5, line 3, line 1, central, line 2, line 4 and line 6). The amplitude of the induced activity was analyzed with the same factors for evoked activity in a different interval (120–220 ms). For the induced activity, another temporal window (220–800 ms) was checked with the factors described previously.

#### 2.4.3. Gamma Band

In this case, we performed the same ANOVAs described in the alpha section. To analyze the latency of evoked and induced gamma activities, one ANOVA with the following two variables was performed: group factor (levels HC and MS patients) and activity factor (levels: evoked and induced activity). For the amplitude variable, we performed two ANOVAs (evoked and induced activities) with the same factors and levels applied for alpha ANOVAs. However, the intervals were different for evoked and induced activity. For the evoked activity, the amplitude was analyzed in the 90–160 ms interval after the onset stimulus (target or standard). For the induced activity, the interval used for analysis was 90–130 ms.

In all the statistical analyses described, sphericity was corrected with Greenhouse–Geisser, and a statistically significant result was considered at *p* < 0.05. Post hoc analyses were performed using Bonferroni correction.

## 3. Result

### 3.1. Behavioural Data

The analysis showed differences between groups in reaction time [F (1,29): 25.247; *p* < 0.001; ŋ^2^: 0.465]. The healthy controls (mean: 338.08; SD: 40.62) were faster than the MS patients (mean: 394.05; SD: 38.25). Regarding accuracy, healthy controls (mean 96.66; SD: 5.31) showed more precision than the MS patients (mean 78; SD: 26.42) [U: 135.50; *p* < 0.001; ŋ^2^: 0.320]. The reaction time and accuracy values are presented in Table 2. In an exploratory analysis no correlation was found between EDSS scores and behavioral data.

### 3.2. Alpha Band

In latency, we observed differences between groups (HC and MS) [F (1,58): 14.552; *p* < 0.001; ŋ^2^: 0.312] (Figure 2). In addition, the post hoc Bonferroni comparison confirmed that the healthy controls were faster than the MS patients in both evoked (*p* = 0.034) and induced activities (*p* < 0.001). The latency and amplitude values are shown in Table 3. Additionally, the activity factor was statistically significant [F (1,58): 8.301; *p* = 0.005; ŋ^2^: 0.262] with evoked activity being faster than induced activity. No correlation was found between EDSS scores and spectral activity of alpha band.

**Figure 3:**
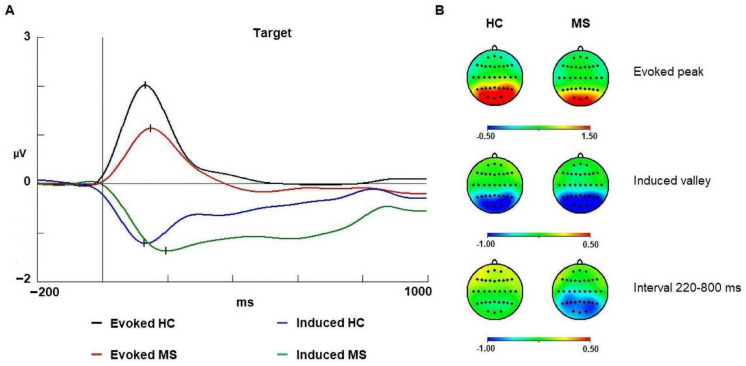
Spectral activity of alpha band in both groups. (**A**) Evoked and induced waves for the alpha band (8–13 Hz) in the visual oddball task. (**B**) 2D head maps for the peak and valley latencies of evoked and induced activity. Abbreviations: MS: multiple sclerosis; HCs: healthy controls; ms: milliseconds; µV: microvolts.

Regarding amplitude in the evoked activity, the healthy controls showed a larger amplitude than that of the MS patients [F (1,58): 9.390; *p* = 0.003; ŋ^2^: 0.220]. In the group x anterior–posterior interaction, post hoc Bonferroni comparison showed that the posterior regions (parieto-occipital) of healthy controls were larger than those of MS patients (*p* = 0.008). In the induced activity, the ANOVA did not find amplitude differences between groups in the 120–220 ms interval. However, in the 220–800 ms interval after onset of stimulus, the statistical analysis showed that the MS patients reached a higher decrement of induced activity than that of the healthy controls in the group factor [F (1,58): 6.562; *p* = 0.013; ŋ^2^: 0.177] (Figure 3).

### 3.3. Gamma Band

Regarding latency, we did not observe differences between groups (HC and MS) (*p* = 0.158). The latency and amplitude values are shown in Table 3. Similar to the alpha band, the activity factor for the gamma band showed significant differences [F (1,58): 16.142; *p* < 0.001; ŋ^2^: 0.376]. In this case, the evoked activity (mean: 122; SD: 36) was also faster than the induced activity (mean: 139; SD: 45).

**Figure 4:**
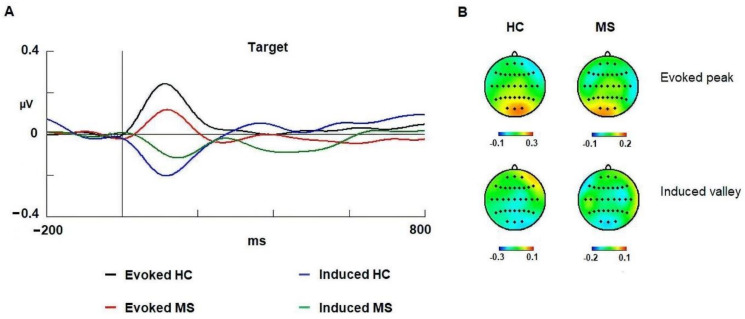
Spectral activity of gamma band in both groups. (**A**) Evoked and induced waves for the gamma band (30–45 Hz) in the visual oddball task. (**B**) 2D head maps for the peak and valley latencies of evoked and induced activity. Abbreviations: MS: multiple sclerosis; HCs: healthy controls; ms: milliseconds; µV: microvolts.

Regarding the amplitude, there were no differences between groups in the 90–160 ms interval for the evoked activity (*p* = 0.121) or in the 90–130 ms interval for the induced response (*p* = 0.062) (Figure 4). In an exploratory analysis no correlation was observed between EDSS scores and spectral activity in gamma band.

## 4. Discussion

Some studies have applied the visual oddball task in patients with MS [5,6,9,10,12,49]. Few authors have compared the performance between MS patients and healthy controls [9,10,49]. In two of these previous studies, no differences in reaction time in the visual oddball paradigm were observed between MS patients and healthy controls [9,10]. However, we found a delay in reaction time in MS patients compared to healthy controls. As we suggest below, this delay may be explained by general slowing, impaired preparation for a response to the onset of the target or both. Regarding accuracy, we observed a poorer performance in MS patients than in healthy controls, suggesting that there was no speed–accuracy trade-off in this experiment. Therefore, these results indicated a cognitive deficit in the sample of patients participating in the study.

### 4.1. Evoked Alpha

In evoked alpha, an increase (synchronization) in activity was observed in both groups (Figure 3). This increase following stimulus onset has also been described in the visual oddball task [25,47] and in other paradigms, such as ANT [20]. Some authors have suggested that evoked alpha activity at latencies within the 100–200 ms interval represent the spectral content of early ERPs (P1 and N1) [16,18,19]. In contrast, we observed differences between groups in the latency at which the maximal synchronization of alpha activity was reached. Healthy controls showed faster latencies than patients with MS (Table 3). The most plausible cause for the longer latency may be the demyelination that MS patients suffer [1]. It is well known that MS pathology can provoke a delay in transmission within a network or between neural networks, causing cognitive deficits [50].

Another alternative interpretation may be related to preparatory activity. Some authors have suggested that a fixed SOA can generate expectations in the absence of cues [51,52]. In this way, subjects can develop accurate expectations about the onset of the stimulus even in the absence of temporal and/or spatial cues. However, a previous study has reported a poor expectative preparation response in MS patients even in paradigms with temporal and spatial cues [53]. In addition, Liu et al. [54] demonstrated that those who show a poorer level of alertness are worse in the temporal estimation of the onset of the stimulus. In fact, diverse studies have described an impairment in alertness for MS patients [55,56,57,58]. Therefore, the delay in latency observed in MS patients may be facilitated by deficits in alertness and poorer expectative mechanisms.

Regarding amplitude, the analyses showed significant differences between groups (Figure 3). Healthy controls reached higher amplitude (synchronization) than did patients with MS. This larger amplitude in healthy controls may represent a better performance in early sensory processing of the stimulus compared to the MS patients [59]. The lower amplitude in MS may be due to a deficit in the neural recruitment of neuronal firing causing a lower level of synchronization.

Atrophic processes may also be responsible for a change in the topography of the spectral modulations and subsequently responsible for a lower amplitude. In fact, MS patients exhibited a lower amplitude in the parieto-occipital regions compared to the healthy control group. However, the electrode with maximum amplitude in both groups was p4p, and no substantial change in the general distribution over the scalp for the evoked modulation was present in the current data. This reasoning is in accordance with previous studies, in which atrophy measures and EDSS scores (cognitive measures included) do not correlate in the first stage of the disease [60].

### 4.2. Induced Alpha

In the induced activity, a decrease (desynchronization) in the alpha band was observed as in previous studies [47,61] (Figure 3). This decrement (valley) has been associated with the need to reduce alpha to allow other cognitive processes, operating in alpha or in other frequencies, to perform their function [16,25,36,62]. With regard to induced activity, the healthy controls reached the maximum desynchronization (decrease) faster than the MS patients, suggesting that the healthy controls can decrease the induced alpha activity to allow a more efficient cognitive processing than the MS patients. As for the evoked response, demyelination and/or impaired mechanisms of expectation may be responsible for this delay.

Surprisingly, no significant differences were found in the maximum amplitude reached in either group in the valley of the induced activity (Figure 3); this outcome contradicts previously studies that have reported alterations in the decrement of the alpha band in MS patients [20,35,36]. However, MS patients showed a greater decrease in induced alpha activity than healthy controls in the 220–800 ms interval (after valley). A plausible interpretation is that the first decrement of alpha was similar in this task because the cognitive requirements of the task may not have reached the level involved in other cognitive tasks such as the ANT [20]. However, the decrement of induced alpha activity needs to be maintained in longer latencies (220–800 ms) to allow the patient to perform the task as best as possible [20,35,36].

### 4.3. Evoked Gamma

In the current experiment, we observed a positive evoked gamma modulation in similar latencies compared to previous studies [63]. Interestingly, the data showed no differences in latency or amplitude between groups (Figure 4). Therefore, based on these results, we suggest that the MS patients did not manifest alterations in the mechanisms indexed by the early gamma-evoked response as previously described [20].

### 4.4. Induced Gamma

Some studies have related gamma activity to the translation of attentional focus [20,28,33,64]. Vázquez-Marrufo et al. [20] observed an increase (synchronization) in the amplitude of the induced activity in conditions whereby translation of attentional focus is demanded. Moreover, this increase in amplitude was greater in MS patients, which suggests that patients compensated for their deficits by recruiting a larger number of neurons to translate the attentional focus in a spatial location.

The aim of this work was to provide further evidence for the role attributed to induced gamma activity in a previous study [20]. Because the current visual oddball task with a central presentation of the stimuli did not imply a considerable translation of attentional focus, we expected no increase (synchronization) in the induced activity or a difference between the two groups. The oscillations depicted in Figure 3 show a small desynchronization at approximately 90–130 ms, which may be caused by other mechanisms of gamma band modulation that are not impaired in the MS group.

### 4.5. Evoked and Induced Activity

Finally, the diverse results from this study provide more evidence to previous studies that evoked and induced responses are dissociable and represent different cognitive mechanisms. The first evidence is related to the different latencies observed for evoked and induced responses (faster for the evoked response) in the first modulation caused by the presentation of the stimuli. Moreover, the induced response may have lasted longer than the evoked response. Lastly, there were differences in amplitude between groups for the longer latencies in the induced activity but not for the evoked modulation. Together, these results indicate that induced modulation represents a linked mechanism to the evoked response, representing a reduction in neural noise that competes with the sensory processing of the stimuli represented by the evoked response but is also related to other cognitive processes that operate later in information processing.

## 5. Conclusions

We conclude that MS patients manifest a cognitive impairment shown by the behavioural parameters, which may be partially due to alterations in the evoked and induced spectral responses. Moreover, the present study demonstrated that both activities are segregated in time, which confirms a different role in the current task and a long latency cognitive impairment for MS patients. Lastly, the comparison of these results and those observed previously with a different paradigm [20] suggest that gamma-induced activity is strongly related to the translation of attentional focus.

## Figures and Tables

**Table 1:** Sociodemographic data.

	MS	HC
Sex (m/f)	8/22	11/19
Age (years, mean ± SD)	41.5 ± 9.04	37.43 ± 10.68
Handedless (left/right)	3/27	3/27
Duration of disease (years, mean ± SD)	8.46 ± 4.3	-
EDSS (mean, range)	2.95 ± 1.6	-

Abbreviations. MS: Multiple Sclerosis; HC: Healthy Controls; SD: Standard deviation; EDSS: Expanded Disability Status Scale; m: male; f: female.

**Table 2:** Behavioral results.

Reaction Time			Accuracy		
Group	Healthy Controls	Multiple Sclerosis	Subject	Healthy Controls	Multiple Sclerosis
Mean	338	394	Mean	96	78
SD	40.6	38.2	SD	5.3	26.4
*p*-value	<0.001		*p*-value	<0.001	

The *p*-value refers to the “group” factor difference (see text for details). Reaction time and accuracy were measured in milliseconds and percentages, respectively. Abbreviations: SD: standard deviation.

**Table 3:** Evoked and induced values for alpha band (8–13 Hz) and gamma band (30–45 Hz).

**Alpha Band**							
**Latency (mean ± SD)**				**Amplitude (mean ± SD)**			
	**HC**	**MS**	***p*-value**		**HC**	**MS**	***p*-value**
**Evoked**	132 ± 32	167 ± 54	0.034	**Evoked**	2.15 ± 0.83	1.30 ± 1.05	0.003
**Induced**	136 ± 37	189 ± 60	<0.001	**Induced**	–1.33 ± 0.60	–1.57 ± 1.22	0.292
**Gamma Band**							
**Latency (mean ± SD)**				**Amplitude (mean ± SD)**			
	**HC**	**MS**	***p*-value**		**HC**	**MS**	***p*-value**
**Evoked**	117 ± 37	126 ± 36	1.000	**Evoked**	0.275 ± 0.2	0.142 ± 0.16	0.058
**Induced**	126 ± 41	152 ± 46	0.098	**Induced**	–0.246 ± 0.14	–0.17 ± 0.15	0.067

Latency and amplitude were measured in milliseconds and microvolts, respectively. Abbreviations: MS: multiple sclerosis; HCs: healthy controls; SD: standard deviation.
